# Formal and Informal Learning and First-Year Psychology Students’ Development of Scientific Thinking: A Two-Wave Panel Study

**DOI:** 10.3389/fpsyg.2017.00133

**Published:** 2017-02-10

**Authors:** Demet Soyyılmaz, Laura M. Griffin, Miguel H. Martín, Šimon Kucharský, Ekaterina D. Peycheva, Nina Vaupotič, Peter A. Edelsbrunner

**Affiliations:** ^1^Department of Psychology, Istanbul Bilgi UniversityIstanbul, Turkey; ^2^Faculty of Film, Art and Creative Technologies, Dún Laoghaire Institute of Art, Design and TechnologyDún Laoghaire, Ireland; ^3^Faculty of Psychology, Pontifical University of SalamancaSalamanca, Spain; ^4^Faculty of Psychology, Ghent UniversityGhent, Belgium; ^5^Department of Psychology, University of AmsterdamAmsterdam, Netherlands; ^6^Department of General, Experimental and Genetic Psychology, Sofia University St. Kliment OhridskiSofia, Bulgaria; ^7^Department of Psychology, University of LjubljanaLjubljana, Slovenia; ^8^Research on Learning and Instruction, Department of Humanities, Social and Political Sciences, ETH ZurichZurich, Switzerland

**Keywords:** epistemic cognition, informal learning, need for cognition, self-efficacy, scientific thinking, psychology students

## Abstract

Scientific thinking is a predicate for scientific inquiry, and thus important to develop early in psychology students as potential future researchers. The present research is aimed at fathoming the contributions of formal and informal learning experiences to psychology students’ development of scientific thinking during their 1st-year of study. We hypothesize that informal experiences are relevant beyond formal experiences. First-year psychology student cohorts from various European countries will be assessed at the beginning and again at the end of the second semester. Assessments of scientific thinking will include scientific reasoning skills, the understanding of basic statistics concepts, and epistemic cognition. Formal learning experiences will include engagement in academic activities which are guided by university authorities. Informal learning experiences will include non-compulsory, self-guided learning experiences. Formal and informal experiences will be assessed with a newly developed survey. As dispositional predictors, students’ need for cognition and self-efficacy in psychological science will be assessed. In a structural equation model, students’ learning experiences and personal dispositions will be examined as predictors of their development of scientific thinking. Commonalities and differences in predictive weights across universities will be tested. The project is aimed at contributing information for designing university environments to optimize the development of students’ scientific thinking.

## Introduction

Scientific thinking encompasses purposeful thinking with the aim to enhance knowledge, using the abilities to generate, test and revise theories as well as being able to reflect on how knowledge is acquired and changed (Kuhn, 2002). It is a prerequisite for engagement in scientific inquiry (Kuhn et al., 2000). Little is known about the development of scientific thinking during aspiring researchers’ development, especially at the early undergraduate level (Parent and Oliver, 2015).

The 1st-year at university is a particularly critical educational period for students’ development of skills, interests, and aspirations (Jenert et al., 2015). For those students who want to become researchers, 1st-year education gives a first impression of science, with core courses found in psychology programs such as research methods and statistics (Stoloff et al., 2009). These educational experiences contribute to students’ future motivation and understanding of science (Manning et al., 2006; Jenert et al., 2015). Not all students know in their 1st-year of university education if they want to engage in research and become researchers. But if they decide so at any point in the future, it is necessary to equip them with advanced scientific thinking as early as possible. Thus, the quality of 1st-year education can influence students’ further aspirations and development as students and researchers. In this study, we examine the influence of learning experiences in 1st-year psychology on students’ development of scientific thinking. One aim of the present study is to identify learning experiences related to the development of scientific thinking in the 1st-year of higher education as well as to pinpoint those that are most prevalent among successful scientific thinkers. This way, we try to capture the experiential profile of budding psychology researchers. Such findings could be of vital service in the development of psychology curricula that better reflect the learning needs of aspiring researchers as well as motivate students to become such.

A facet of scientific thinking that we consider central for potential future researchers is scientific reasoning^1^. Scientific reasoning delineates the skills needed to conduct scientific inquiry, such as argumentation, drawing inferences from data, and engaging in experimentation (Zimmerman, 2007). It includes understanding and identifying relevant variables and how to interpret the information obtained from an experiment and various other research designs (Klahr, 2000).

Related to scientific reasoning skills, students should understand basic statistical concepts to evaluate the strength and uncertainty of scientific evidence. Previous research has shown that misconceptions about common statistical indicators, such as *p*-values and confidence intervals, are prevalent in student and teacher populations (Hoekstra et al., 2014; Morey et al., 2015), and in published research literature (Gelman and Stern, 2006; Nieuwenhuis et al., 2011). It is not surprising that students develop misconceptions about statistics, given that classical statistical methods violate common sense (Wagenmakers, 2007; Duffy, 2010). Psychology students commonly learn about null hypothesis significance testing in their statistics courses, which leads them to make interpretations based on arbitrary *p*-values previously set by researchers. This method violates common sense and leads to misunderstandings because the results are actually based on observations that have not occurred (Wagenmakers, 2007). For example, researchers intuitively tend to think that hypotheses tests inform them about the probability that the alternative hypothesis is true, but hypotheses tests based on *p*-values have a different aim; they only inform about the long-term frequency of possible data given the null hypothesis. Crucially, those misconceptions can lead to wrong inferences, both in the conduct of research but also in the evaluation of published literature, which might have contributed to a current crisis in the confidence of psychological science (Pashler and Wagenmakers, 2012). With this in mind, we regard the absence of statistics misconceptions as a relevant aspect of scientific thinking in today’s psychology students as developing researchers.

Another core facet of scientific thinking is epistemic cognition, which encompasses beliefs about knowledge, knowing, and the processes by which those beliefs are formed and influence further learning (Hofer and Pintrich, 1997; Kitchener, 2002; Greene et al., 2008). Greene et al. (2010) developed a model of epistemic and ontological cognition that integrates prior models by positing positions and dimensions. Each of four positions (Realism, Dogmatism, Skepticism, and Rationalism) corresponds to a distinctive pattern of individuals’ beliefs along the three dimensions of simplicity and certainty, justification by authority, and personal justification of knowledge (Hofer, 2000). Simple and certain knowledge refers to the opinion that knowledge is isolated, simple and constant over time, justification by authority reflects a belief that knowledge can be ambiguous but holds greater weight when presented by an authority figure, and personal justification is a belief that all information presented must be engaged with critically before judging it to be true, and even then, it may not remain true over time. The first developmental stage, realism, represents strong beliefs in simple and certain knowledge, justification by authority, and personal justification. The position of dogmatism is demonstrated through strong justification by authority. The position of skepticism reflects strong personal justification. Lastly, rationalism presents moderate agreement with justification by authority and personal justification, but strong disagreement with the concept of simple and certain knowledge. Epistemic cognition can influence critical thinking, scientific argumentation, and learning (Kuhn et al., 2000; Nussbaum et al., 2008; Franco et al., 2012), and it is related to students’ self-regulated learning (Bråten and Strømsø, 2005). Several studies thus far have linked epistemic cognition with students’ learning achievements. A study conducted by Muis and Franco (2009) illustrates that epistemic cognition directly influences achievement goals of students in educational psychology course, which, in turn influenced their engagement in their tasks and final course achievement. In the similar vein, Bråten and Ferguson (2014) showed that epistemic beliefs contribute to achievement over and above cognitive capacity and personality traits of students. Moreover, Chen et al. (2014) showed that students who are self-efficacious about learning science, approach a task by examining arguments from several sources to make a decision, thus indicating a moderating role of self-efficacy in how epistemic cognition is related to academic outcomes. The results of these studies therefore support the idea that epistemic cognition plays a major role in students’ further engagement and development of scientific thinking and we consider it a facet of scientific thinking that should develop early in psychology students.

The development of scientific thinking begins in the first stages of life and continues throughout childhood and adolescence up into adulthood (Sodian et al., 1991; Kuhn et al., 2000, 2015; Zimmerman, 2007). However, this development does not occur automatically but through steady input from deliberate learning experiences (Kuhn, 2002, 2009; Klahr et al., 2011). Perhaps the most conspicuous way of improving the level of scientific reasoning is through formal education. In fact, research shows that students with higher level of education as well as students who were exposed to a research methodology course are more likely to exhibit better scientific reasoning skills (Lehman and Nisbett, 1990; Lawson et al., 2000). Demonstrating and engaging students in quantitative methodology and scientific inference improves their skills in theoretical modeling and experimentation (Duschl and Grandy, 2013; Holmes et al., 2015). Further learning experiences that predict university students’ scientific thinking include collaborative learning (e.g., Gokhale, 1995; Johnson et al., 1998), social media use (Ebner et al., 2010; Dabbagh and Kitsantas, 2012; Kassens-Noor, 2012; Vivian et al., 2014), taking research methods and statistics courses (Lehman and Nisbett, 1990; VanderStoep and Shaughnessy, 1997), passive and active participation in research projects (Wayment and Dickson, 2008; VanWormer et al., 2014) and taking laboratory modules that include interpreting the results of an experiment (Coleman et al., 2015). Thus, university environments offer varied experiences that can help students develop scientific thinking.

In order for students to develop scientific thinking, it is not sufficient that relevant learning opportunities are offered at university. It is necessary that students show high engagement in formal activities and beyond. In the current study, we therefore look into students’ engagement in relevant formal and informal learning activities. Informal learning at university can be defined as self-directed learning in the sense that the student chooses the topic, curriculum, and contents, and learning and assessment modalities, with the aim to develop knowledge, skills, or competences (Hofstein and Rosenfeld, 1996; Laurillard, 2009). It is closely related to conceptions of student engagement (Krause and Coates, 2008), as well as self-sustained, self-initiated, and free choice learning (Falk, 2001; Barron, 2006; Yang, 2015). Based on this definition, examples of informal learning experiences are attending science conferences, reading scientific books, and engaging in science-related discussions with peers. Formal learning, in comparison, is highly structured through university bodies in its curriculum, fixed learning activities, and assessment, with a course achievement or qualification as an end product (Resnick, 1987; Eshach, 2007; Patrick, 2010). This distinction posits informal learning as interest-driven, in comparison to formal learning as curriculum-based, assessment-driven, and qualification-oriented activities.

What factors predict students’ engagement in learning activities that are likely to foster their scientific thinking? A characteristic that we take into account is students’ science self-efficacy, that is, the confidence they have in their ability to do science (Beißert et al., 2014). Self-efficacy, defined as the belief in own capability to succeed (Bandura, 1997), is a major predictors of university students’ cognitive engagement, academic persistence in science-related courses, and career choices (Chemers et al., 2001; Walker et al., 2006; Chen and Usher, 2013). Along with self-efficacy, need for cognition has been shown to predict academic success (Elias and Loomis, 2002). It is a stable tendency to engage in and enjoy effortful thinking (Cacioppo and Petty, 1982). Need for cognition is related to intellectual engagement and positive attitudes toward effortful tasks, and thus with a richer personal history of gaining knowledge on a variety of topics (Woo et al., 2007).

In the current study, we assess psychology students twice to examine the contributions of formal and informal learning experiences to their development of scientific reasoning including statistics misconceptions, and epistemic cognition during the 1st-year of study. The assessments take place at the beginning and again at the end of their second semester. We aim to examine interrelations in the development of scientific reasoning and epistemic cognition during the semester, and the contribution of students’ engagement in both types of learning experiences to this development. This includes their additive effects, and the involvement of students’ self-efficacy and need for cognition in these effects. Our definition of informal learning posits that it is self-guided and goes beyond the mere aim of finishing courses and obtaining grades. It thus related informal learning strongly with interest-driven student engagement. Student engagement in educationally purposeful activities is positively related to academic outcomes in 1st-year students as well as students’ persistence at the same institution (Kuh et al., 2008). Similarly, student engagement has been linked to desirable learning outcomes such as critical thinking and academic achievement (Carini et al., 2006). Whereas engagement in formal learning could stem from internal or external motivational factors, engagement in informal activities represents only intrinsically motivated behavior, which is derived from interest and performed for pleasure and desire. For these reasons we assume that informal learning contributes to students’ development of scientific reasoning and epistemic cognition, beyond formal learning. The overarching aim of the design is to establish the circumstances under which potential future researchers in psychological science are able to develop scientific thinking during the early stages of their studies. We therefore examine specific patterns of scientific thinking and its predictors in students who identify themselves as aspiring researchers.

## Materials and Methods

### Design and Sample

The study has a two-wave correlational panel design. Participants will be drawn from the 1st-year psychology courses of 11 universities from eight countries across Europe. We collaborate with 1st-year professors from each university. The choice of universities was based on personal affiliations and on the aim of gathering students from diverse backgrounds across Europe. Psychology student cohorts at the universities span between 40 and 700 students. Students from eight universities will participate during a regular class lesson and the remaining three universities online. At three of the universities, students will receive assessment credits for participating in the study.

Sample size planning based on power analysis is not relevant because we will use Bayesian estimation and hypothesis testing for statistical analysis (Etz et al., 2016; Wagenmakers et al., 2016). In this statistical framework, power is not conceptualized because hypothesis testing is not based on an inferential framework but on continuous evaluation of evidence (Schönbrodt and Wagenmakers, 2016).

### Materials and Equipment

#### Choice of Measures

For every construct that we aim to assess, a literature search was done in the PsycInfo and Scopus databases to identify available measures. The choice of the instruments was based on psychometric quality, appropriateness for university context, administration time, translation feasibility, and meaningfulness of usage in a variety of international universities. Regarding psychometric quality, we ensured that basic analysis such as factor analysis, estimation of reliability or internal consistency had been conducted and achieved at least moderate results. Appropriateness for the 1st-year of university was taken into account insofar as we tried to estimate on which level Psychology students develop during their 1st-year. For example, scientific reasoning is a broad construct, and we chose an instrument that assesses skills which we think are critical for students’ further development, and likely to show at least some development already during their first university year. The chosen instrument assesses principles of experimental design that we deem relevant for understanding the critical quality characteristics of any research the students learn about (Drummond and Fischhoff, 2015).

#### Demographics Questionnaire

Students’ demographic characteristics will include their age, gender, former university education, career aspirations, grades in high school, the grade of first university examination and family socioeconomic status (see Appendix A). For the latter, we will ask students about their parents’ highest achieved education, bedroom availability and the number of books at home in their adolescence (Evans et al., 2010). Socioeconomic status is assessed to examine its influence on the main study variables and to estimate other variables’ influence while controlling for it. We will assess family socioeconomic status because university students are still in education, which constrains their own educational level and also their working situation, the most common indicators of personal socioeconomic status. Family socioeconomic status is thus commonly assessed for research in academic contexts (Caro and Cortes, 2012). Students’ estimated score from the first principal component of the four variables will be used as an indicator of their family socioeconomic status. Finally, we will assess the quantity of formal education relevant for developing scientific thinking (number of methodology and statistics-related courses, number of philosophy of science and epistemology-related courses).

#### Scientific Reasoning

As a measure of scientific reasoning, the *Scientific Reasoning Scale* developed and validated by Drummond and Fischhoff (2015) will be used. It contains eleven true or false items in which hypothetical research scenarios are described and the participant has to decide whether the scenario can lead to proposed inferences. Each of the items relates to a specific concept crucial for the ability to come to valid scientific conclusions. The concepts include understanding the importance of control groups and random assignment, identifying confounding variables, and distinguishing between correlation and causation. Scores on the SRS show adequate internal consistency (Cronbach’s α = 0.70) and correlate positively with cognitive reflection, numeracy, open minded thinking, and the ability to analyze scientific information (Drummond and Fischhoff, 2015). Following this scale, we added an additional item assessing students’ understanding of sample representativeness (Appendix B). Students’ mean score on the scale will be used in descriptive analysis as an indicator of their scientific reasoning. Whether the item on sample representativeness can be added to the scale will be decided based on a confirmatory factor analysis: It will be added in case its factor loading is within the range of the other items.

#### Statistics Misconceptions

We developed a questionnaire encompassing five questions that deal with common statistical misconceptions (Appendix B). Items dealing with *p*-value and confidence interval misinterpretations were taken directly from Gigerenzer (2004) and Morey et al. (2015). We chose the item with the highest prevalence of wrong answers among university students from each article to achieve high variance in our sample of 1st-year students. We further developed items similar in structure dealing with the interpretation of non-significant results, the equivalence of significant and non-significant results (Gelman and Stern, 2006; Nieuwenhuis et al., 2011), and sample representativeness. The items share structure and answer format with the scientific reasoning scale by Drummond and Fischhoff (2015). We added the items after the end of the scientific reasoning scale. Participants are also asked whether they have ever learned about *p*-values, confidence intervals, and sample representativeness. In case they check “no,” their answers on the respective questions will be treated as missing values. Students’ mean value across the four questions dealing with *p*-values and confidence intervals will be used as an indicator of their statistics misconceptions. The question on sample representativeness, as described above, will be used as an additional item of the scientific reasoning scale.

#### Validation Questions

For the Scientific Reasoning Scale and the added statistics misconceptions items, we will add one open-answer validation question. Each student will receive the following question at one random item of the 16 items that the two scales encompass: “*Why did you choose this answer? Please provide an explanation*.”, followed by two lines on which the students are supposed to provide a short rationale for their multiple choice-answer. The question to which this additional open answer is added will differ randomly between students, so that a random subsample of the students will deal as validation sample for each question. We implement this validation measure because the SRS to the best of our knowledge has not yet been translated into our sampled languages and not been used in the sampled countries. It is therefore necessary to examine whether 1st-year psychology students’ answers on these questions reflect the target construct. The statistics misconceptions to the best of our knowledge have not yet been thoroughly validated but rather used to assess the prevalence of wrong answers among students and academics, and we developed three of the questions on our own, therefore we include them in this validation procedure.

#### Epistemic Cognition

To assess epistemic cognition we will administer the Epistemic and Ontological Cognition Questionnaire (EOCQ; Greene et al., 2010). It contains 13 items and a 6-point item response scale ranging from 1 (completely disagree) to 6 (completely agree). The instrument takes into account the contextuality of epistemic cognition by providing the opportunity to insert a domain into the item stems (Greene et al., 2008). We insert *Psychology* and *Psychological science* for the domain that the students should rate the items about. Five items represent simple and certain knowledge (example: “*in psychological science, what is a fact today will be a fact tomorrow*”), four items represent justification by authority (“*I believe everything I learn in psychology class*”), and four items represent personal justification (“*in psychological science, what‘s a fact depends upon a person’s view*”). Higher ratings of ten items indicate stronger beliefs and high ratings of three items indicate weaker beliefs. Reliability estimates (*H* coefficient) range from 0.45 to 0.90 depending on facet and context (Greene et al., 2010). Mean scores on all three subscales will undergo mixture modeling analysis, which will yield an epistemic cognition-profile for each student that will be used for further analysis (Greene et al., 2010).

#### Need for Cognition

We will use the Need for Cognition Short Scale (NFC-K; Beißert et al., 2014) to measure the tendency to engage in and enjoy thinking. The short scale is a modified 4-item version of the 18-item Need for Cognition Scale created by Cacioppo and Petty (1982). On a 7-point scale the students are asked to rate to which extent they agree with four simple statements. An example item is “*I would prefer complex to simple problems.*” Mean scores from this scale will be used for descriptive analysis, with higher scores indicating that students are more motivated to apply their thinking skills. Test retest reliability is *r* = 0.78, Cronbach’s α = 0.86 (Beißert et al., 2014). The score will be used to predict students’ development of scientific thinking, and also as a control variable to examine which variables predict students’ development beyond need for cognition.

#### Science Self-Efficacy

The Science Self-Efficacy (SSE) scale, which consists of 10-items used by Moss (2012) will be used (Cronbach’s α > 0.80). It is a modified version of a vocational self-efficacy survey designed by Riggs et al. (1994). It particularly aims to measure confidence in skills to engage in scientific inquiry. The items are rated on a scale from 1 to 10 (1 = *not able or not true at all*, 10 = *completely able or completely true*). An example item is “*I have all the skills needed to perform science tasks very well.*” Students’ mean score on the scale will be used for statistical modeling. The score will be used to predict students’ development of scientific thinking, and also as a control variable to examine which variables predict students’ development beyond science self-efficacy.

#### Formal and Informal Learning Experiences

We developed a survey to assess students’ engagement in learning experiences that we presume relevant for the development of scientific thinking (Appendix C). The selection of experiences is based on the discussed literature, and it will be further informed and adapted based on the pilot study interviews (Appendix D). Our definitions of formal and informal learning imply a continuum of formality within and across learning activities. For example, a frequent formal learning activity is the studying of a text that is mandatory reading for a research methods course. When students gain interest in the text contents, they might initiate further voluntary reading to inform themselves beyond the course requirements, which in our definition is then an informal learning experience. Our assessment method encompasses a wide variety of prescribed and non-prescribed scientific learning experiences: For each of the assessed activities that can be either formal or informal, we ask students how often they engaged in these as part of mandatory course activities, or for reasons going beyond these. Specifically, we let students rate subjectively for experiences where this applied how much they engaged in them because it was obligatory for course requirements (formal engagement), because it was obligatory but they were also interested (formal and informal engagement), or merely out of own interest (informal engagement).

In the second part of the survey, we ask students about the most relevant three courses they took that were related to research methods, statistics, science, history of science or other similar concepts. We ask for up to three courses because we studied the official bachelor curricula from the targeted universities and most students will not have more highly relevant courses during their first and second semester. Therefore, reporting on further courses might make it strongly subjective which courses the students deem relevant to this question, and it might take rather long and be exhausting to report details on any relevant courses they could think of. To check that they did not have many more relevant courses we, however, ask in the demographics for the absolute numbers of relevant courses. Thus, for up to three most relevant courses, they first list the names of the courses and whether the courses were mandatory or elective. Then, we ask students about their general engagement in these courses (student presence, devoted working time), and course quality (ratings of overall course quality, teaching quality, frequency of inquiry and reflective course elements). Finally, reflecting informal engagement, they rate how much they engaged in each of these courses out of their motivation or interest, beyond the course requirements. Estimating principal components, we will weigh general course engagement across courses with course content ratings to yield an indicator of formal engagement, and informal (out of own motivation or interest) engagement with course quality ratings to yield an indicator of informal engagement.

### Translations and Pilot Study

Considering students from different countries’ levels of competence in English may not be sufficiently high, the materials and instruments have been translated into Spanish, Slovenian, Turkish, Bulgarian, and Czech by the local researchers from these countries. Then, they have been back translated by bilingual speakers to enable reconciliation of the translated texts with the original. During a pilot study, the materials and instruments were administered to a small number (10 from each country) of 2nd–4th year psychology students with cognitive surveying and interviewing to identify problematic passages in terms of ambiguous or confusing instructions and translations (Ziegler et al., 2015). During the cognitive surveying, participants were asked to read the instructions and items aloud. After each passage, they were instructed to report everything that came to their mind when thinking about the instruction or item and what they were thinking while answering the items. In the end, they were asked to reflect freely on the purpose, comprehensibility, and quality of the instrument. This data were used to alter potential problematic passages. Proposed changes were again translated back to English for comparison with the original. Pilot participants were also requested to respond to several interview questions regarding their formal and informal educational experiences throughout their lives that they believe might have contributed to their scientific reasoning and epistemic cognition (Appendix D). Their responses served to improve the formal and informal experiences survey, so that it would more adequately reflect students’ relevant learning experiences.

### Stepwise Procedures

The data will be collected at two time points. The first assessment will be conducted during the first 2 weeks of the second semester (between January and March) and the second will take place within the last 2 weeks of the academic schedule before exams (May and June), depending on each university’s calendar. For universities at which collaborators agree to in-class assessments, these will take place directly in the classrooms or other provided university space. Professors will be asked to reward students with course credits for research participation, depending on the ethical policy of the institution. Ideally, with the professors’ prior consent, the entire first-year courses will be assessed during a lecture. The local researchers in each country will distribute the questionnaires before the assessment starts and collect them afterward. In case an in-class administration of our instruments is not possible, we will ask the students to participate in an online version of our assessments. An online version has been prepared in the Qualtrics (Qualtrics, Provo, UT, USA) environment with a similar structure to the pen and paper version. For the online version, students will be provided with a hyperlink and encouraged to fill it in at their convenience within a week. If they have not yet finished the survey, they will receive a reminder email 2 days before this time limit.

**FIGURE 1:**
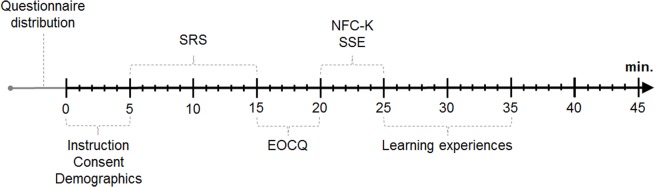
**Structure of the main assessments.** From left to right: Time in minutes. *SRS*, Scientific Reasoning Scale, including statistics misconceptions and one open validation question; *EOCQ*, Epistemic and Ontological Cognition Questionnaire; *NFC-K*, Need for Cognition Short-Scale; *SSE*, Science Self-Efficacy.

The questionnaires will have the same structure in the in-class and in the online version. In both cases, participants will be given a short explanation of what the research is for, and what their participation will entail, which will be read aloud by the experimenter in class. They will then be asked to read an information sheet and read and sign a consent form, prior to proceeding with the assessment. One administration process is expected to last for about 35 min. The scales more strongly related to cognitive skills will be presented in the beginning of the assessment and the learning experiences will be assessed in the end to prevent the experiential questions from influencing later answers. The structure of the assessments is depicted in **Figure 1**.

The participants will first be asked to compose an identification code consisting of their mother’s and father’s initials, and the month that they were born (in mm format). They will then be asked to complete the demographics information about themselves. They will subsequently proceed to complete five scales measuring scientific reasoning, statistical misconceptions, epistemic cognition, need for cognition, and scientific self-efficacy. This will reflect their skills and attitudes after one semester studying psychology. In addition, they will be asked to complete the survey regarding their formal and informal learning experiences during the first study semester. In the end of the assessment, the students will be thanked for their participation, and informed that the assessment will take place again in the end of the semester. They will be asked to refrain from discussing the assessment with each other or to look up the contents.

At the end of the second semester, participants will be approached as before to participate in the study. They will be asked to write their identification number as before, and to again complete the same scales as at the first assessment. On the demographics sheet, this time they will be asked additionally whether and to which extent they discussed the contents of the first assessment with peers or looked up the contents. The survey on formal and informal learning experiences will this time be referring to experiences during their second study semester. At this point participants will be given a debriefing form and thanked for their participation.

## Proposed Analysis And Anticipated Results

### Qualitative Data Analysis

Qualitative data will stem from the pilot study interviews. Transcriptions of the interviews will be analyzed using content analysis. The analysis will be aimed at identifying relevant formal and informal learning experiences else than those known from available literature. Insights from these data will be used to refine the learning experiences questionnaire for the main assessments.

We will also analyze the open validation questions about scientific reasoning and statistical misconceptions to see whether the correct multiple choice-answers on the items reflect the intended concepts (Drummond and Fischhoff, 2015). Given the fact that the items are forced choice between true or false, we of course expect that some of the correct answers will be a result of guessing. We will encode whether the rationale for the answer is sufficient to come to the correct choice given the specific question. Then, we will try to group answers that did not have the correct rationale for the answer to see common misunderstandings. This will inform us about the validity of the items. We will also seek for common misconceptions leading to erroneous answers and try to categorize them with open followed by axial coding to get insight into why students make mistakes regarding the specific concepts. This will inform us what aspects of given concepts are hard to grasp and which misconceptions should be deliberately targeted by university lecturers.

### Confirmatory Statistical Analysis

**FIGURE 2:**
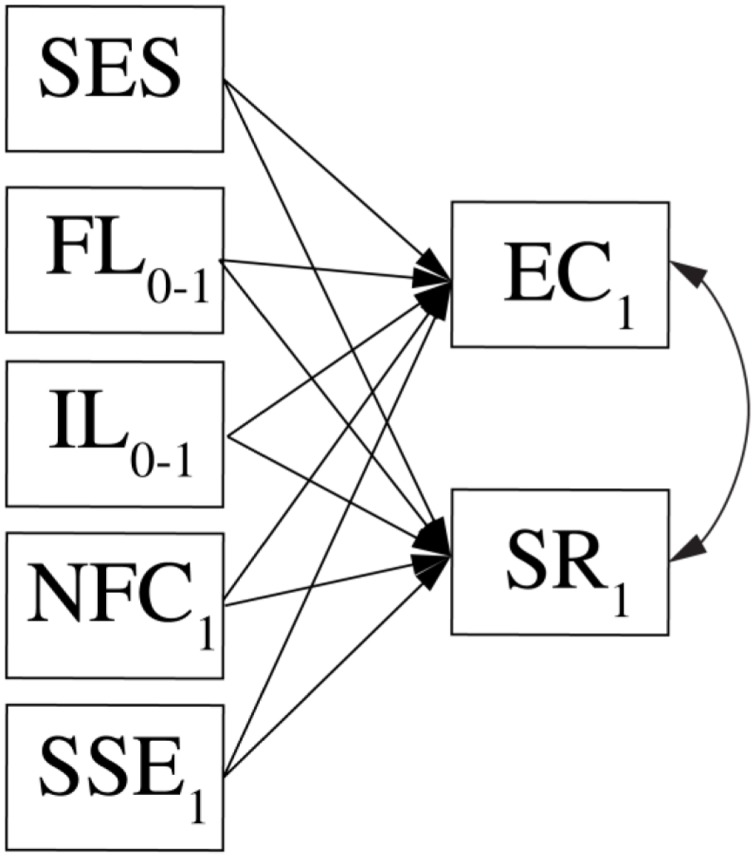
**Depiction of the cross-sectional model in which students’ scientific thinking in the beginning of the second semester is predicted from their learning experiences, personal characteristics, and socioeconomic status.** *SES*, family socioeconomic status; *FL0-1*, formal learning experiences during first semester; *IL0-1*, informal learning experiences during first semester; *NFC*, need for cognition at first assessment; *SSE*, science self-efficacy at first assessment.

Bayesian structural equation modeling will be applied to examine our main research questions. The models will be written using the r2jags (Su and Yajima, 2012) and rjags (Plummer, 2013) packages in the R software (R Core Team, 2013) to be estimated in the JAGS software (Plummer, 2003). There will be two main models. In a cross-sectional model, we will predict students’ scientific thinking and research aspirations after the first half year from their educational experiences during the first half year, science self-efficacy and need for cognition, and from their learning experiences and family socioeconomic status. A depiction of the structural relations in this model is provided in **Figure 2**.

**FIGURE 3:**
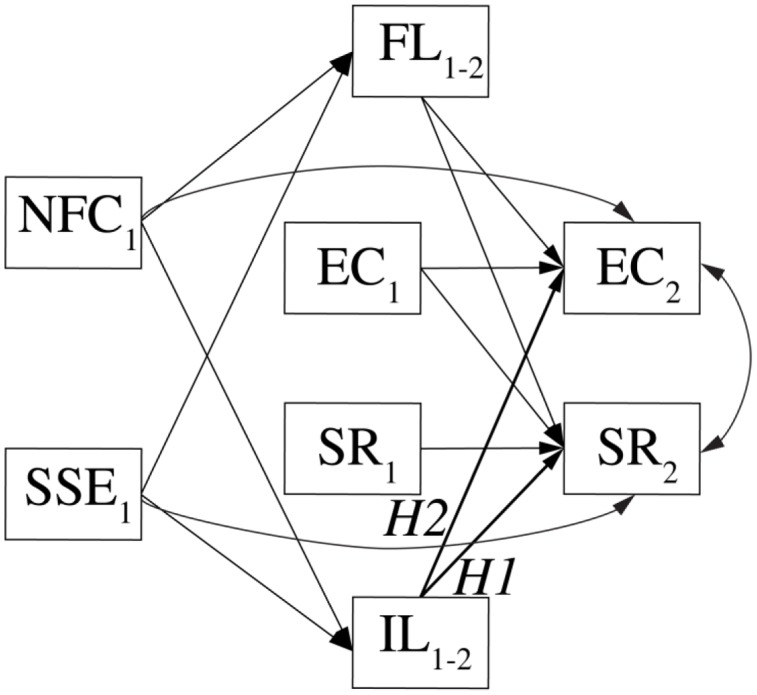
**Longitudinal model in which the change in students’ epistemic cognition and scientific reasoning from the beginning (*EC1, SR1*) to the end (*EC2, SR2*) of the second semester will be predicted.** *NFC1*, need for cognition at first assessment; SSE1, science self-efficacy at first assessment; FL1-2, formal learning during second semester; IL1-2, informal learning during second semester. Paths predicted based on main hypotheses are bold and marked as *H1* and *H2*.

In a longitudinal model, we will examine developmental interrelations between students’ scientific reasoning and epistemic cognition, and predict their development from students’ formal and informal learning experiences during the second half year, and how these are influenced by students’ personal characteristics. A depiction of the structural relations in this model is provided in **Figure 3**.

Bayes factors will be used for hypothesis testing. The tested predictions based on our hypotheses include that informal learning experiences predict the development of scientific reasoning and epistemic cognition, controlling for formal learning experiences, need for cognition, and science self-efficacy.

The models will be estimated separately for students from each university to examine commonalities and differences in predictive weights. Since sample sizes vary strongly between countries and institutions, samples from some universities might not be sufficiently big to ensure convergence and precision of model estimation. Data from the biggest samples will therefore be used to slightly inform the parameter priors from the smaller samples. Only priors of non-focal (i.e., non-hypothesis-testing) parameters will be informed in this way (for an overview of related techniques, see McElreath, 2016, pp. 424–430). This strategy is similar to hierarchical modeling but implies weaker partial pooling. Scripts for all confirmatory analyses will be uploaded to the Open Science Framework prior to data analysis. We will interpret the magnitude of obtained Bayes factors based on the accruing samples from the different universities. The Bayes factors will be computed as a ratio of likelihoods of two models that describe the theoretical alternatives we put to the test (Jarosz and Wiley, 2014). We derive those models for two focal hypotheses (depicted in the **Figure 3** as H1 and H2) here. Given we control for formal learning experiences (FL in **Figure 3**), need for cognition (NFC), and science self-efficacy (SSE), we expect that parameters for informal learning experiences (IL in **Figure 3**) have a positive value. This expectation is equivalent to a one-sided hypothesis. We will therefore use so called one-sided hybrid Bayes factors (Morey and Rouder, 2011). As a null model, we will combine a point nil with part of the Cauchy distribution from the range of values of 0 up to the point where the effect size becomes important (equivalence region). As an alternative model, we will use the remaining of the Cauchy distribution. The Cauchy will have a scaling factor of 0.5, the equivalence region will be defined as <0,0.1> and the mixture probability of the two parts of the null model will be equal to 0.5 (the point nil and equivalence region will have the same weights). This type of Bayes factors have been shown to possess desirable properties (Morey and Rouder, 2011). They asymptotically converge toward support for null or alternative if the true parameter lies in the area of one of the respective models, and remain indifferent if the true parameter lies on the boundary of null and alternative (0.1 in this case).

Missing data stemming from attrition or single non-answered items will be dealt with in the Bayesian analysis. Specifically, students’ self-reported interest in research and becoming researchers and all other study variables that might be associated with participation willingness and missingness will be used to estimate students’ missing data (see McElreath, 2016).

### Exploratory Statistical Analysis

These analyses serve mainly to find specific patterns between all variables and trying to identify students interested in becoming future researchers. In addition, they will serve to develop potential hypotheses about profiles of psychology students interested in becoming researchers. For this research question, we do not have specific hypotheses and we will use exploratory analyses to examine how the intention to become a researcher or not is associated with the other study variables. Specifically, we will use two methods. We will use network modeling to explore relations between the main study variables at the two assessments. For this analysis the mgm-package will be used, which can handle different distributions of the exponential family and applies regularization for sparse solutions (Haslbeck and Waldorp, 2016). The estimated networks at the two assessments will provide a concise and informative overview of interrelations between the study variables in the beginning and end of students’ second semester. We will also estimate finite mixture models (Hickendorff et al., unpublished), to extract profiles of scientific thinking. We will examine profiles including scientific reasoning, the three epistemic cognition facets, and students’ research aspirations as profile indicator variables, the two dispositional scales as profile predictors, and statistics misconceptions as a distal variable.

To further substantiate comparisons between universities and countries, we will examine measurement invariance, which shows whether the assessment instruments have comparable structure across different samples. Measurement invariance is not of critical importance for our hypotheses but it is informative for exploratory purposes, to see for example whether the instruments function similarly in the different languages. In our Bayesian framework, we will be able to handle small deviations from invariance by modeling approximate invariance (Van De Schoot et al., 2012).

## Limitations

The design will allow estimating the predictive value of formal and informal learning experiences but causal conclusions are not fully warranted for various reasons. Controlling for students’ general maturation in higher education would be possible by adding a control group, for example 1st-year students from a different field. Such a comparison would, however, be biased by self-selection effects because we cannot assign students randomly to different fields of study.

Another limitation is that measurement will take place twice within one semester, specifically within the second semester. This might be early for expecting students to develop in scientific reasoning, epistemic cognition, and also statistical misconceptions, depending on when these topics are part of students’ courses. Not all students might learn about these topics in their 1st-year. We looked into students’ official curricula at the target universities and in all of them there are courses that might be relevant but this is not clear for all universities. Regarding assessing twice within one semester, more change might be expected during a longer time period. We will, however, ask the students also for relevant experiences during the first semester, which overall will yield a picture of the whole 1st-year, which covers the focus of our study.

Also, because the collecting data will take place on two different occasions during the semester, there may be attrition between time one and time two. However, by aiming to collect data in class, we hope to maximize initial potential participation and minimize potential attrition rate by time two. Missing data will also be minimized by ensuring that participants use the same identification code on both occasions. To maximize participation and minimize attrition, assessments will be conducted in-class at most universities. To minimize the missing data due to unintended skipping of the responses within the questionnaires, we will encourage students to thoroughly review their responses to ensure that they answered every question. In the online version, we will use automatized options to check missing responses to alert participants that they did not answer the question.

Another issue regards that we use self-report measures, particularly retrospective measures for students’ learning experiences. These might be biased because remembering and subjectively judging the quality of courses from the last semester is error prone. Averaging ratings across students will hopefully lead to averaging out some error and the magnitude of between-student variance might indicate how error-prone retrospection is in this case. Also, for epistemic cognition it has been pointed out that self-report measures only allow quite superficial assessment (Mason, 2016). Alternatively, the idea of incorporating both quantitative and qualitative research methods upon EC has been supported (e.g., Greene and Yu, 2014). For the aims of our study we deem the EOCQ self-report measure appropriate but it will not allow a comprehensive look into the processes underlying students’ development.

With reference to the country comparisons, even if it is not our main focus, they might be biased by the fact that at some universities all students participate within courses, at some they participate voluntarily without incentive, and at some voluntarily with incentive. Consequently, we acknowledge that these differences in recruitment may influence the results. In fact, voluntary participation in this research topic about scientific thinking can be seen as an indicator of student engagement and interest in science (and then, be categorized as an informal experience). In addition, it could happen that samples where students participate voluntarily with no incentive are overrepresented in the levels of informal experiences’ engagement. We will check to which extent students’ research interest differs between these three groups.

Finally, since our research question is aimed at 1st-year students, we assess scientific thinking on a level that might be seen as quite basic for a higher education level. Measures on a more advanced level could be added, for example assessing justification in multiple sources as another facet of epistemic cognition (Ferguson and Bråten, 2013). More advanced measures might add relevant information, but they might potentially also mostly reveal floor effects due to 1st-year students’ limited experiences.

## Ethics Statement

The study materials and consent forms have been developed in accordance with ethical norms and guidelines from all participating countries and universities. The study was approved by the Institutional Review Board of Istanbul Bilgi University and IADT Institute Research Ethics Committee.

## Author Contributions

All authors listed, have made substantial, direct and intellectual contribution to the work, and approved it for publication.

## Conflict of Interest Statement

The authors declare that the research was conducted in the absence of any commercial or financial relationships that could be construed as a potential conflict of interest.

## Footnotes

1 Scientific reasoning is sometimes used interchangeably with scientific thinking (and sometimes with the related but broader construct critical thinking, Halpern et al., 2012) but in the context of the present research we deem it useful to conceptualize scientific reasoning and epistemic beliefs as facets of scientific thinking.
