# Supplementary material for: Formal and Informal Learning and First-Year Psychology Students’ Development of Scientific Thinking: A Two-Wave Panel Study
Source: Front Psychol. 2017 Feb 10;8:133. doi: 10.3389/fpsyg.2017.00133 (PMC5301459; doi:10.3389/fpsyg.2017.00133)
Supplement: Supplementary file 1 [file Data_Sheet_1.docx]

**Appendix A - Demographics**

- Please provide the following to assign you an anonymous code:

Initial of your mother’s first name: [ ] e.g.: Corinne: [C]

Initial of your father’s first name: [ ] e.g.: Devin: [D]

Your day of birth: [ ] [ ] e.g.: 12^th^ of March [1] [2]

- Age:  _________ Gender: _______
- Email (university email if available):  __________________________
- Secondary email (non-university if available):  __________________________

Note:

Your email information will only be used to contact you at maximum twice to ask for your participation in further rounds of this study for which you will be offered financial compensation. If you decline twice you will not receive further emails. This information will not be used in any other way or be given to third parties.

- Did you undergo any prior university education before studying psychology?

No [ ] Yes, for ____ years.

- What is the highest achieved education of your father?

Compulsory school [ ] Vocational education [ ] high school [ ] university degree [ ]

- What is the highest achieved education of your mother?

Compulsory school [ ] Vocational education [ ] high school [ ] university degree [ ]

- Did you have your own bedroom when you were 14?

Yes [ ] No [ ]

- About how many books were there around your family’s house when you were 14 years old?

None [ ]   1 or 2 [ ]   Around 10 [ ]   Around 20 [ ]   Around 50 [ ]

Around 100 [ ]   Around 200 [ ]   Around 500 [ ]   1000 or more [ ]

- What about was your average degree (GPA) in your final high school (secondary school/lissé) examinations?  _____________________
- What about was your average degree (GPA) in your final high school science classes (e.g., Physics, Chemistry)?  _____________________
- What was your grade in your first examination at university? _____________
- Are you currently employed?

No [ ] Yes, approximate hours per week: _____________________

- Do you currently plan to continue and finish your psychology studies?

Yes [ ] No [ ]

- How many courses did you have last semester in which research methods or statistics were addressed?

________

- How many courses did you have last semester in which the philosophy of science, research, or scientific knowledge were addressed?

________

- Are you generally interested in research?

Not at all 0 1 2 3 4 5 Totally

- What are your career aspirations?

___________________________________________________________________

- Which division of psychological science do you find most interesting so far?

_______________________

**Appendix B - Statistics Misconceptions**

1. A researcher wants to test statistically whether there is a difference in the intelligence of male and female students. The result of the statistical test is "p > .05, not statistically significant".

*True or False?* The researcher can conclude that there is no difference in the intelligence of male and female students.

2. A researcher wants to test statistically whether younger and older participants show differences in knowledge gains from before to after undergoing a course. For the younger participants, the statistical result for a test of their knowledge gains is "p < .05, statistically significant". For the older participants, the statistical result is "p > .05, not statistically significant".

*True or False?* The researcher can conclude that there are differences in knowledge gains between younger and older participants.

3. Assume you have a treatment that you suspect may alter performance on a certain task. You compare the means of your control and experimental groups (say 20 subjects in each sample). Further, suppose you use a simple independent t-test and your result is (t = 2.7, d.f. = 18, p = 0.01).

*True or False?* You know, if you decide to reject the null hypothesis, the probability that you are making the wrong decision.

4. A researcher conducts an experiment, analyzes the data, and reports: The 95% confidence interval for the mean ranges from 0.1 to 0.4!

*True or False?* The researcher can conclude that the “null hypothesis” that the true mean equals 0 is likely to be incorrect.

5. An Icelandic researcher created an online survey. The link to the survey got viral on the social media and he was able to obtain over 60,000 responses in a week. Based on this high number, he concluded that the sample can be regarded as representative of the Icelandic population, as the survey sample represents almost 20 % of the entire population.

*True or False?* There are no problems with the researcher's claim about the representativeness of the sample.

*After single random question:*

*Why did you choose this answer? Please provide an explanation*.

___________________________________________________________________________

___________________________________________________________________________

___________________________________________________________________________

**Have you ever learned about p-values?**

Yes [ ] No [ ]

**Have you ever learned about confidence intervals?**

Yes [ ] No [ ]

**Have you ever learned about sample representativeness?**

Yes [ ] No [ ]

*Validity question for the second assessment:*

- **Have you looked up or discussed answers to these questions with your peers?**

Yes [ ] No [ ]

**Appendix C - Learning Experiences Survey ***

A number of common student activities are listed below. Think about the previous semester which has already ended and answer the following as truthfully as you can on a scale from 1 to 4; 1 means “never” and 4 means “very often”.

|  | **Please state approximately how often you engaged in each of these activities for the following reasons:** | | |
| --- | --- | --- | --- |
|  | Because it was obligatory for a course.  Never Very Often | Because it was obligatory for a course but I was also interested.  Never Very Often | Because it was NOT obligatory but I was interested.  Never Very Often |
| Attending lectures about research methods or statistics | 1 2 3 4 | 1 2 3 4 | 1 2 3 4 |
| Attending lectures in which philosophical topics about research or science were discussed | 1 2 3 4 | 1 2 3 4 | 1 2 3 4 |
| Reading psychology or science textbooks | 1 2 3 4 | 1 2 3 4 | 1 2 3 4 |
| Preparing for classes | 1 2 3 4 | 1 2 3 4 | 1 2 3 4 |
| Doing homework | 1 2 3 4 | 1 2 3 4 | 1 2 3 4 |
| Reading scientific articles | 1 2 3 4 | 1 2 3 4 | 1 2 3 4 |
| Reading books on research methods or statistics | 1 2 3 4 | 1 2 3 4 | 1 2 3 4 |
| Discussing scientific issues with peers | 1 2 3 4 | 1 2 3 4 | 1 2 3 4 |
| Discussing scientific issues with lecturers outside classes | 1 2 3 4 | 1 2 3 4 | 1 2 3 4 |
| Doing online courses/MOOCs about research methods, statistics, or science | 1 2 3 4 | 1 2 3 4 | 1 2 3 4 |
| Looking up scientific information about a topic of interest | 1 2 3 4 | 1 2 3 4 | 1 2 3 4 |
| Watching science documentaries | 1 2 3 4 | 1 2 3 4 | 1 2 3 4 |
| Following scientific discussions in blogs/forums | 1 2 3 4 | 1 2 3 4 | 1 2 3 4 |
| Following news about science | 1 2 3 4 | 1 2 3 4 | 1 2 3 4 |
| Analyzing research data | 1 2 3 4 | 1 2 3 4 | 1 2 3 4 |
| Asking questions during/after a lecture | 1 2 3 4 | 1 2 3 4 | 1 2 3 4 |
| Discussing scientific issues with researchers | 1 2 3 4 | 1 2 3 4 | 1 2 3 4 |

A number of common student activities are listed below. Think again about the previous semester, which has already ended, and answer the following as truthfully as you can.

In the previous semester, did you…

...attend a scientific conference? No [  ] Yes [  ]

...present something at a scientific conference? No [  ] Yes [  ]

...do a research-related internship? No [  ] Yes [  ]

In the previous semester, how many times did you…

...act as research participant? _____ times

...conduct a research study within a course? _____ times

...conduct a research study outside of obligatory curricula?_____ times

...attend scientific exhibitions? _____ times

...give oral presentations? _____times

...do academic writing about course contents or studies others conducted? _____ times

...do academic writing about your own studies or thoughts about research? _____ times

| Please list the three most relevant courses from last semester that addressed research methods or statistics, or the history, theory, or philosophy of research, science, or scientific knowledge. |
| --- |
| Course titles:  ***Course 1:*** _____________________________________________________  ***Course 2:*** _____________________________________________________  ***Course 3:*** _____________________________________________________  Were any of these a voluntary/elective course?  No [ ] Yes, Course ______  Did you attend all or most of the lectures?  ***Course 1:*** Yes [ ] No [ ] ***Course 2:*** Yes [ ] No [ ] ***Course 3:*** Yes [ ] No [ ]  About how much time did you devote overall to course work, lecture and exam preparations?  ***Course 1:*** ____hours ***Course 2:*** ____hours ***Course 3:*** ____hours  To which extent did the courses cover research methods such as research design or statistics?  ***Course 1:*** *Not at all* 0 1 2 3 4 5 *A lot*  ***Course 2:*** *Not at all* 0 1 2 3 4 5 *A lot*  ***Course 3:*** *Not at all* 0 1 2 3 4 5 *A lot*  Did the courses cover the history, theory, or philosophy of research, science, or scientific knowledge?  ***Course 1:*** *Not at all* 0 1 2 3 4 5 *A lot*  ***Course 2:*** *Not at all* 0 1 2 3 4 5 *A lot*  ***Course 3:*** *Not at all* 0 1 2 3 4 5 *A lot*  How do you rate the overall quality of the courses?  ***Course 1:*** *Not good*  0 1 2 3 4 5  *Very good*  ***Course 2:*** *Not good*  0 1 2 3 4 5  *Very good*  ***Course 3:*** *Not good*  0 1 2 3 4 5  *Very good*  How do you rate the teaching in the courses?  ***Course 1:*** *Not good*  0 1 2 3 4 5  *Very good*  ***Course 2:*** *Not good*  0 1 2 3 4 5  *Very good*  ***Course 3:*** *Not good*  0 1 2 3 4 5  *Very good*  Did the teaching in the courses involve reflective discussions?  ***Course 1:*** *Not at all* 0 1 2 3 4 5 *A lot*  ***Course 2:*** *Not at all* 0 1 2 3 4 5 *A lot*  ***Course 3:*** *Not at all* 0 1 2 3 4 5 *A lot*  How much did you engage in the course contents (e.g., discussions with peers, looking up further information) **out of your own interest, beyond the course requirements**?  ***Course 1:*** *Not at all* 0 1 2 3 4 5 *A lot*  ***Course 2:*** *Not at all* 0 1 2 3 4 5 *A lot*  ***Course 3:*** *Not at all* 0 1 2 3 4 5 *A lot*  Which grades did you receive?  ***Course 1:*** *___*  ***Course 2:*** ___  ***Course 3:*** ___ |

**Appendix D - Pilot Interview**

**Information regarding the tape recording sessions**

Your participation in this interview study will be used to inform research on the development of psychology students. We are taping the following interview for our research purposes. The audio taped will be transcribed to a text by the local researcher ____________ and this text will be analyzed anonymously for the research project.

**Data protection and confidentiality**

The submitted information will be stored in a way that no other person can have access to this data addressing in strict compliance with the relevant local legislation*.*

Under no circumstances personal data will be published, guaranteeing the confidentiality and rigid compliance of the professional secrecy while managing the information obtained. Your personal identity will be related to the study results only based on your participant’s number. The audio taped will be destroyed 5 years after publication of the research.

**Withdrawal of consent**

If at any moment you would like to stop participating in the interview, please let us know and you can withdraw from the interview immediately.

**Statement of consent**

I, ___________________________________ declare to have read, understood and accepted the terms described in this informed consent. I consent to participate in the study and I am aware that the data derived from the interview will be used to fulfill the research objectives.

_______________, __/__/__

**Introduction:**

I am part of a research project in which we are interested in the development of psychology students’ scientific thinking. This means we look into psychology students’ views and beliefs about what science is, what they think about psychology as a science and about scientific knowledge, and what they know and think about research methods.

For the beginning, I would like you to share your general thoughts on these topics related to your studies. Please start reflecting on these topics and tell me everything you are thinking about. The first few questions will be very broad and open. There is nothing specific that you have to mention; please just tell me everything that comes to your mind. Do you have any questions before I start with the first question?

**Part 1: Interview Questions
Section 1: Current Thoughts and Formal and Informal Experiences in Psychology and Science**

1. How did you become interested in (studying) psychology? (Brief response)
   1.1 Follow-up question if not mentioned: “Why did you start or choose to study psychology”?

1.2 Follow up-question if not mentioned: “What is your aim; what do you want to do with your finished studies in Psychology?”

**Section 2: Epistemic Beliefs and Scientific Reasoning**

2. What do you think about your skills in research methods and statistics?

2.1 What do you think has influenced your skills in research methods and statistics? This can include attendance and work for university courses but also other things that you did out of your own interest.

2.2 After the description of each experience, ask first: For a more detailed description: “Could you elaborate further on how this might have shaped your scientific Thinking?”

2.3 To focus on university:

“You have described mostly experiences outside of regular university courses. Could you describe any relevant experiences that took place within your regular university courses”

2.4 To focus on informal experiences:

“You have described mostly experiences within the attendance or work that you had to do to finish your regular university courses. Can you think of other experiences that went beyond course requirements, for example things you engaged in out of your own interest?”

2.5 If extra prompting is required:

“With experiences outside the context of regular university courses, I mean experiences like chats with your colleagues, going to non-mandatory talks or workshops, reading books, reading on the internet, and similar experiences. Can you think of any such or related experiences that might have shaped your scientific thinking?”

or

2.6 “With university courses, I mean courses like research methods, statistics, history and philosophy of science, or something similar. Can you think of any such or related courses that might have shaped your scientific thinking?”

3. How did you learn about the theory and philosophy behind research in psychology?

**Section 3: Formative Experience in Childhood and Adolescence**

4. Can you think of any experiences in your childhood and adolescence that influenced your understanding of science in any way, in school and outside of school?

4.1 If they skip either childhood or adolescence completely: Can you think of anything else in your *childhood/adolescence* (ask the one they did not mention by themselves)?

**Section 4: Final question:**

6. Did you ever engage in research on your own, for example at high school, at university, or at other occasions?

6.1. Follow up if yes: How do you think did this experience shape your scientific thinking?

7. How strongly are you interested in becoming a researcher, on a scale from one to ten? One stands for not at all, ten stands for very strongly.

7.1. What influenced this level of motivation to become or not become a researcher?

7.2. Did this level of motivation change since you entered the psychology Degree?

**Part 2: Check for comprehension**

- 1. What did you think of these surveys?
  2. Was there anything unclear? Is there any way that this could be made more clear?
  3. What do you think we want to assess with these surveys?

**Part 3. Follow-up interview after checking the surveys**

The two main surveys:

Scientific reasoning (SRS) and epistemic cognition (EOCQ).

8. Now you have seen the survey where you had to state whether researchers’ inferences were correct or wrong. I am interested in where your knowledge about such aspects of research methods comes from. Is there anything that helped you to think in this way or learn about these things? That could again be university courses but also any other experiences in and out of your university life.

9. You have also seen the survey where you had to agree or disagree with statements about scientific knowledge and truth in Psychology. I am interested in where your beliefs about these things come from. Is there anything that helped you to think in this way or learn about these things? That could again be university courses but also any other experiences in and out of your university life.
